# The complete chloroplast genome of *Prunus campanulata* ‘Fugui’ (Rosaceae)

**DOI:** 10.1080/23802359.2022.2106796

**Published:** 2022-08-26

**Authors:** Zheng-Feng Wang, Xue-Cheng Yang, Ming-Xuan Zheng, Qing Zhou

**Affiliations:** a Guangdong Provincial Key Laboratory of Applied Botany, South China Botanical Garden, Chinese Academy of Sciences, Guangzhou, China; b Southern Marine Science and Engineering Guangdong Laboratory (Guangzhou), Guangzhou, China; c Center for Plant Ecology, Core Botanical Gardens, Chinese Academy of Sciences, Guangzhou, China; d College of Forestry and Landscape Architecture, South China Agricultural University, Guangzhou, China

**Keywords:** Chloroplast, genome assembly, next generation sequencing, *Prunus campanulata*

## Abstract

*Prunus campanulata* ‘Fugui’ is newly bred cultivar. Here, we report its complete chloroplast genome. The length of the *P. campanulata* ‘Fugui’ chloroplast genome is 157,948 bp, with a large single-copy region of 85,948 bp, a small single-copy region of 19,128 bp and a pair of inverted repeat regions of 26,436 bp each. The genome contains 90 protein-coding genes, 65 transfer RNA genes and 9 ribosomal RNA genes. In addition, the genome contains 67 simple sequence repeats. Phylogenetic analysis revealed that *P. campanulata* ‘Fugui’ is genetically related to previously reported *P. campanulata*.

*Prunus*, a genus of flowering shrubs and trees in the rose family (Rosaceae). It includes more than 400 species worldwide, but only distribute in northern temperate regions. Many of *Prunus* species are economically important, including almond, apricot, cherry, peach and plum. Cherry trees have high ornamental values and renowned for their blossoms in all branches. *Prunus campanulata* (Maxim.) Yu et Li 1883 is one of most charming cherry trees, native to China, Japan and Vietnam. Due to low reproductive barrier, it has many cultivars by breeding. *Prunus campanulata* ‘Fugui’ is newly bred cultivar with pink double flower in China. It has stable morphological traits since cultivated and is considered as an acceptable *Prunus campanulata* cultivar in the market. Due to its beautiful flowers, it is highly demanded and now has been widely planted in southern China as ornamental plant (Supplemental Figure S1). However, due to unknown parentage breeding history, its origin is obscure. To better understanding the origin of *P*. *campanulata* ‘Fugui’ in the future, therefore, we report its complete chloroplast genome to provide a genomic resource for the phylogeny study.

**Figure 1.**
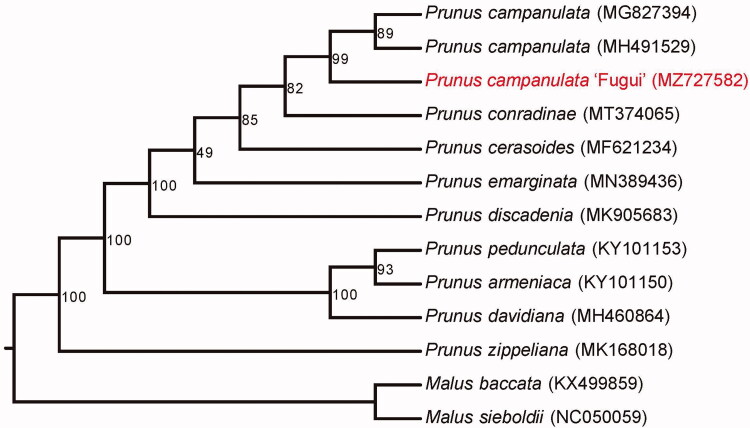
Maximum-likelihood phylogenetic tree for *Prunus campanulata* ‘Fugui’ and 12 additional species. The GenBank accession numbers are shown in parentheses. The numbers on the branches are bootstrap supported values.

Fresh leaves of *P. campanulata* ‘Fugui’ were collected from the South China Agricultural University (N23° 09′ 34", E113° 20′ 23″). A voucher specimen was deposited at the Herbarium of South China Agricultural University (Ming-Xuan Zhen, zhengmx@scau.edu.cn) under the voucher number 32204. The genomic DNA of *P. campanulata* ‘Fugui’ was extracted by the CTAB (cetyltrimethylammonium bromide) method. Using a 2 × 150 bp paired-end sequencing strategy, the extracted DNA was sequenced using the Illumina HiSeq X Ten system (Illumina, San Diego, CA) and about 26 Gb whole- genome sequencing reads were produced. The sequences were then used to assemble the chloroplast genome of *P. campanulata* ‘Fugui’ by NOVOPlasty 4.3.1 (Dierckxsens et al.^2017^). The assembled genome was then annotated with CPGAVAS2 (Shi et al. 2019), GeSeq (Tillich et al. 2017) and PGA (Qu et al. 2019). The annotated genome is now available in GenBank under the accession number MZ727582. Maximum-likelihood phylogenetic analysis was perform in PhyloSuite 1.2.2 (Zhang et al. 2020) and RAxML 8.2.12 (Stamatakis 2014) with the concatenated protein sequences of 73 chloroplast coding genes for *P. campanulata* ‘Fugui’ and the other 12 species. Both *Malus baccata* and *Malus sieboldii* were used as outgroups. The simple sequence repeat (SSR) was identified with MISA-web (Beier et al. 2017). The substitution hotspots estimated by nucleotide diversity (*Pi*) in the three *P. campanulata* chloroplast genomes (Figure 1) were calculated using DnaSP v6.12.03 (Rozas et al., 2017) by a window size 500 bp and step size 250 bp.

The *P. campanulata* ‘Fugui’ chloroplast genome was 157,948 bp in length with the GC content of 36.72%. The genome showed a large single-copy region of 85,948 bp, a small single-copy region of 19,128 bp and two copies of inverted repeat regions of 26,436 bp each. After annotation, a total of 137 genes were identified in the *P. campanulata* ‘Fugui’ chloroplast genome, including 90 protein-coding genes, 65 transfer RNA genes and 9 ribosomal RNA genes. Phylogenetic analysis revealed that *P. campanulata* ‘Fugui’ was genetically related to previously reported *P. campanulata* (Figure 1).

By aligning with the other *P*. *campanulata* (MG827394 and MH491529) chloroplast genomes, 63 single nucleotide polymorphisms (SNPs) and 42 insertion and deletions (indels) were identified among *P. campanulata* ‘Fugui’ chloroplast genome. Furthermore, a total of 67 simple sequence repeats (SSR) were discovered in *P. campanulata* ‘Fugui’ genome. These SSRs included 63 mononucleotides (A/T/G/C), 4 dinucleotides (TA/AT). Compared to the other two *P*. *campanulata* chloroplast genomes, that both displayed 66 mononucleotides and 4 dinucleotide type SSRs, *P. campanulata* ‘Fugui’ genome contained less mononucleotide SSRs than them, but the same dinucleotide SSRs to them. *Pi* values ranged from 0.0000 to 0.0120, among them three regions showed high value great than 0.005 within the sites of 42755–43259 bp, 78780–79563 bp and 109602–110351 bp, representing as hotspots in the genome. These regions contained *rps*2, *ropC*2, *ndhK*, *ndhC* and *rpl*14 genes.

## Supplementary Material

Supplemental Material

## Data Availability

The genome sequence data that support the finding of this study are openly available in GenBank of NCBI at [https://www.ncbi.nlm.nih.gov] (https://www.ncbi.nlm.nih.gov/) under the accession number MZ727582 and is also accessible at https://doi.org/10.13140/RG.2.2.16436.78722. The associated BioProject, SRA, and Bio-Sample numbers for reads are PRJNA752956, SRR15371665 and SAMN20668218 respectively.
