# Supplementary figures and images for: The complete chloroplast genome of Prunus campanulata ‘Fugui’ (Rosaceae)
Source: Mitochondrial DNA B Resour. 2022 Aug 26;7(8):1534–5. doi: 10.1080/23802359.2022.2106796 (PMC9423843; doi:10.1080/23802359.2022.2106796)

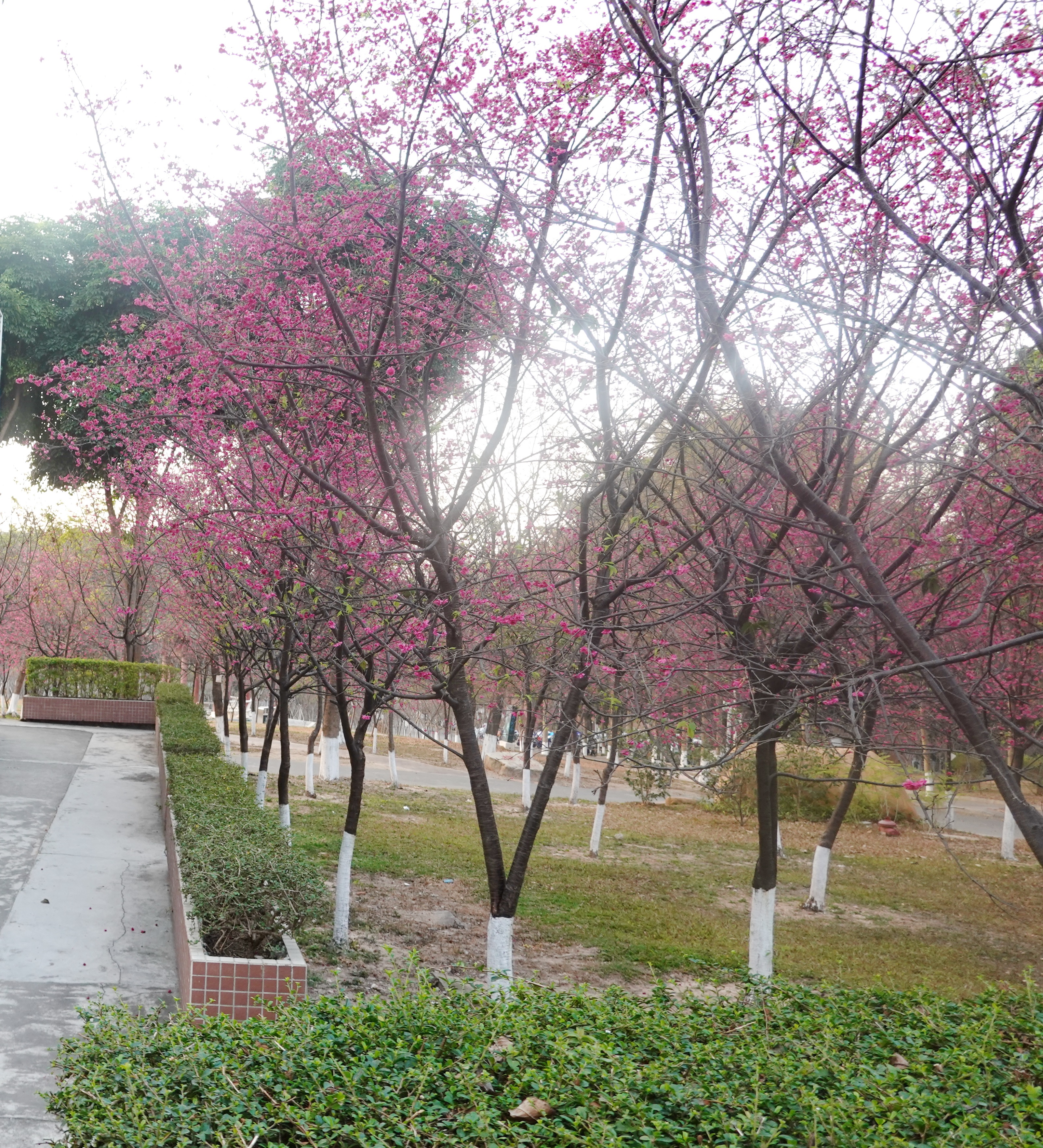

Supplement: Supplemental Material [file TMDN_A_2106796_SM4763.jpg]
